# Reframing early health technology assessment through a lifecycle lens: Commentary on “defining early health technology assessment: building consensus using Delphi technique”

**DOI:** 10.1017/S0266462325100548

**Published:** 2025-10-07

**Authors:** Ramiro Gilardino, Nicole Mittmann, Debjani Mueller, Franz Pichler

**Affiliations:** 1 School of Public Health, University of Buenos Aires Faculty of Medicine, Buenos Aires, Argentina; 2 Canada’s Drug Agency - L’Agence des medicaments du Canada, Ottawa, Canada; 3 Health Technology Assessment Unit, University of Pretoria, Pretoria, South Africa; 4 Health Technology Assessment International, Edmonton, Alberta, Canada; 5 Confluence Health Consulting, Sydney, NSW, Australia

**Keywords:** health technology assessment, lifecycle, drug Innovation, health policy

## Abstract

The article “Defining Early Health Technology Assessment: Building Consensus Using Delphi Technique” offers a useful definition of early HTA (eHTA) as an assessment to inform development, research, or investment decisions. From a lifecycle HTA (LC-HTA) perspective, we emphasize that eHTA should be viewed as part of a broader, continuous evidence and decision-making process rather than a stand-alone activity. Integrating eHTA within LC-HTA strengthens alignment across phases of technology development, supports anticipatory evidence planning, and promotes adaptive reassessment over time—enabling more coherent, learning-oriented HTA systems.

Recent efforts to refine the definition and boundaries of early health technology assessment (eHTA) reflect a growing recognition of the evolving, dynamic nature of health innovation, and decision making. The article entitled “Defining Early Health Technology Assessment: Building Consensus Using Delphi Technique” contributes to a valuable stakeholder-informed definition of eHTA as “a health technology assessment conducted to inform decisions about subsequent development, research and/or investment by explicitly evaluating the potential value of a conceptual or actual health technology” (1). This positions eHTA as a methodological activity designed to inform decisions at the early stages of technology development, where flexibility in design, research planning, and investment strategy is greatest. Importantly, although this definition highlights the methodological contribution of eHTA, it also underscores its role in shaping upstream decision making rather than serving as an isolated academic exercise.

The definition helps distinguish eHTA from related concepts, which is valuable. Yet, to maximize its impact, it may be helpful to frame eHTA within the broader lifecycle perspective that underpins health technology assessment (HTA) practice over time.

As members of the Lifecycle HTA (LC-HTA) Task Force convened by the Health Technology Assessment International (HTAi) Global Policy Forum, we offer this commentary to contextualize the proposed eHTA definition within the lifecycle HTA continuum, highlight where it aligns and diverges, and recommend directions for integration and implementation.

## The evolution of eHTA: Narrow method or lifecycle entry point?

The authors define early eHTA as an HTA conducted to inform decisions about subsequent development, research, and/or investment by explicitly evaluating the potential value of a conceptual or actual health technology (1), focusing largely on development-stage interventions. Although this precision offers useful parameters, it may underrepresent the complexity of decisions that evolve across a product’s lifecycle and the ecosystems in which they operate.

The outputs of the LC-HTA Task Force conceptualizes HTA as a *sequenced process of evidence generation and use*, acknowledging the importance of decision making at various stages not only before launch but also during market entry, reassessment, and disinvestment (2). eHTA, from a lifecycle perspective, is not a siloed activity, rather it serves as an entry point into a more dynamic, adaptive HTA system: one that accommodates evidence needs to evolve and be revisited over the lifetime of the technology.

In our operationalization framework (3), we stress that technologies enter overall HTA processes under uncertainty, often with immature data, shifting comparators, or evolving clinical standards. From our perspective, *eHTA is therefore valuable, not only for binary investment decisions but also as a planning tool to map uncertainties, prioritize data collection, and align evidentiary expectations with regulators and payers.*

## Overlap and complementarity: Methodological considerations

The eHTA definition positions it as a means to apply specific methods (e.g., modeling, scenario analysis, or preference elicitation) in early development phases. We agree that such techniques are central but urge recognition that *methodology alone does not define purpose or scope.*

From our lens, the same methods may apply at multiple stages of the lifecycle. For example, early modeling can support not only value-based pricing assumptions prelaunch but also guide managed entry agreements, real-world data collection priorities, support patient needs, or eventual reassessment. Rather than focusing on novelty, what distinguishes eHTA is the timing, local processes, and specific application of methods within the development pathway. Seen this way, eHTA represents an early entry point into a continuum of HTA-related activities that extend across the lifecycle of a technology, supporting better alignment between evidence generation and decision making over time. Furthermore, there is a need to ensure consistency and interoperability of HTA methods across lifecycle stages among HTA organizations. Decisions made early using simplified or immature assumptions must be traceable and updatable through iterative HTA, creating an evidence continuity that enhances transparency and stakeholder trust. We caution against viewing early HTA as a distinct methodological silo.

## The need for integration, not fragmentation

One potential limitation of the eHTA definition lies in its framing as a bounded activity for R&D strategy. Although this is relevant for industry stakeholders, the definition risks excluding public sector, payer, or system perspectives on early engagement. LC-HTA instead advocates for joint problem-framing early on, with explicit multistakeholder engagement to ensure that no perspective is excluded. This is particularly important in areas with high uncertainty, novel pathways, or disruptive potential, where shared understanding of evidence needs and decision contexts can improve alignment, ensuring decision grade outcomes and reduced downstream inefficiencies.

Integration in practice takes place at multiple levels:

- *Across the lifecycle*: ensuring that early analyses are not “standalone” but linked to reassessment, managed access, and disinvestment decisions.
- *Across stakeholders*: involving regulators, HTA bodies, payers, clinicians, and patients in early dialogues to define decision problems and evidence priorities.
- *Across geographies and systems*: where possible, alignment between jurisdictions can reduce duplication of effort and streamline global development, though integration will always need tailoring to local processes and institutional capacities.

And it could be achieved through eHTA-regulatory dialogues, joint scientific advice with HTA bodies, use of early assessment to define real-world data needs and reassessment triggers, and iterative decision-making frameworks that build from early HTA to inform managed access or conditional reimbursement.

Moreover, *environmental sustainability, equity, and innovation value* are increasingly relevant for eHTA topics that benefit from lifecycle framing (4). If we limit eHTA to only R&D portfolio decisions, we risk omitting key health system goals or identifying patient unmet needs.

## Insights from previous workshops and global engagement survey

As part of our task force (TF’s) continued engagement, we organized a workshop at HTAi 2024 Annual meeting, gathering stakeholder feedback on LC-HTA approaches and its operationalization. Key takeaways that support a more integrated eHTA conceptualization include:

- Stakeholders perceive value in early engagement, ensuring that technologies are better aligned with patient needs, healthcare system demands, and regulatory requirements but highlight the need for transparency about how eHTA insights are used in later decisions.
- Frameworks should link early assessment signals with future reassessment or disinvestment. In some cases, early signals may prevent adoption; in others, a sequential approach enables later disinvestment.
- What is considered “early” depends on system capacity and context. In some jurisdictions, eHTA complements structured reassessment, whereas in others, it may simply be the first chance for dialogue or engage with the decision maker or HTA about a technology.

These insights align with the author’s emphasis on eHTA’s promise but reinforce the idea that it is a flexible gateway, not a standalone product.

## Moving forward

We welcome this initiative as a contribution to definitional clarity. However, we encourage researchers and HTA institutions to situate early HTA within a lifecycle perspective to maximize its strategic and system value. Examples to make this unification possible may be:

- *Position eHTA as the front-end phase of LC-HTA*, clarifying its role within the broader lifecycle continuum. Consolidating definitions can help reduce duplication and terminological confusion, while methodological mapping can illustrate how specific techniques (e.g., modeling, scenario analysis, and preference elicitation) are applied at different lifecycle stages.
- *Clarify triggers for eHTA* based on product novelty, patient need, uncertainty level, or decision-making needs and evidence availability, not just the R&D phase.
- Promote interoperability of HTA processes across stages and among organizations, including early and reassessment HTAs, to avoid duplication and misalignment.
- *Support nascent systems* by employing and adapting eHTA as a capacity-building tool and aligning it with other lifecycle-informed planning functions (e.g., horizon scanning, risk-sharing agreements).

Our task force’s operationalization framework offers a practical structure for this integration, organizing HTA across four sequencing domains (3): problem definition, sequencing of activities, optimization criteria, and contextual tailoring.

Within this structure, eHTA can be clearly positioned and linked to downstream activities, avoiding fragmentation and enabling effective resource use. We welcome the opportunity to dive further into effective implementation.

## Conclusion

eHTA has gained renewed attention as decision makers face accelerating innovation, compressed timelines, and persistent data uncertainty. Although the recent study defines a useful boundary for discussion, a lifecycle lens enhances its relevance by connecting early HTA to the broader decision-making continuum. In this sense, eHTA can also serve an anticipatory function – providing early signals about potential value, evidence gaps, or risks that may warrant further development, targeted research, or even reconsideration of adoption. Positioned within lifecycle HTA, this anticipatory role strengthens the alignment of early assessments with future reassessments, managed access, or disinvestment pathways.

Rather than carving out a methodological or conceptual silo, we believe eHTA should be recognized as an essential component of adaptive, sequenced, and context-aware HTA systems. By embracing its anticipatory function within a lifecycle paradigm, researchers, funders, and HTA bodies can better harness eHTA full potential and move toward more sustainable, responsive assessment practices.
